# Prognostic scores and early management of septic patients in the emergency department of a secondary hospital: results of a retrospective study

**DOI:** 10.1186/s12873-021-00547-8

**Published:** 2021-12-07

**Authors:** GianLuca Colussi, Giacomo Perrotta, Pierpaolo Pillinini, Alessia G. Dibenedetto, Andrea Da Porto, Cristiana Catena, Leonardo A. Sechi

**Affiliations:** 1 Division of Internal Medicine and Emergency Medicine Residency Program, Department of Medicine, University of Udine, 1st floor, Building n.8, Piazzale Santa Maria della Misericordia 1, 33100 Udine, UD Italy; 2 Emergency Department, San Antonio Abate Hospital, ASUFC, 33028 Tolmezzo, Italy

**Keywords:** ROC curve, SOFA, NEWS2, APACHE II, SAPS II, Survival analysis

## Abstract

**Background:**

Sequential Organ Failure Assessment (SOFA) and other illness prognostic scores predict adverse outcomes in critical patients. Their validation as a decision-making tool in the emergency department (ED) of secondary hospitals is not well established. The aim of this study was to compare SOFA, NEWS2, APACHE II, and SAPS II scores as predictors of adverse outcomes and decision-making tool in ED.

**Methods:**

Data of 121 patients (age 73 ± 10 years, 58% males, Charlson Comorbidity Index 5.7 ± 2.1) with a confirmed sepsis were included in a retrospective study between January 2017 and February 2020. Scores were computed within the first 24 h after admission. Primary outcome was the occurrence of either in-hospital death or mechanical ventilation within 7 days. Secondary outcome was 30-day all-cause mortality.

**Results:**

Patients older than 64 years (elderly) represent 82% of sample. Primary and secondary outcomes occurred in 40 and 44%, respectively. Median 30-day survival time of dead patients was 4 days (interquartile range 1–11). The best predictive score based on the area under the receiver operating curve (AUROC) was SAPS II (0.823, 95% confidence interval, CI, 0.744–0.902), followed by APACHE II (0.762, 95% CI 0.673–0.850), NEWS2 (0.708, 95% CI 0.616–0.800), and SOFA (0.650, 95% CI 0.548–0.751). SAPS II cut-off of 49 showed the lowest false-positive rate (12, 95% CI 5–20) and the highest positive predictive value (80, 95% CI 68–92), whereas NEWS2 cut-off of 7 showed the lowest false-negative rate (10, 95% CI 2–19) and the highest negative predictive value (86, 95% CI 74–97). By combining NEWS2 and SAPS II cut-offs, we accurately classified 64% of patients. In survival analysis, SAPS II cut-off showed the highest difference in 30-day mortality (Hazards Ratio, HR, 5.24, 95% CI 2.99–9.21, *P* < 0.001). Best independent negative predictors of 30-day mortality were body temperature, mean arterial pressure, arterial oxygen saturation, and hematocrit levels. Positive predictors were male sex, heart rate and serum sodium concentration.

**Conclusions:**

SAPS II is a good prognostic tool for discriminating high-risk patient suitable for sub-intensive/intensive care units, whereas NEWS2 for discriminating low-risk patients for low-intensive units. Our results should be limited to cohorts with a high prevalence of elderly or comorbidities.

## Background

Patients overcrowding in the emergency department (ED) is an important limiting factor for the quality of the medical assistance [1]. Early decision-making based on predictive scores could quickly address patients to the more appropriate intensity level of assistance and, by that, reducing observation time and the waiting list in ED. Sequential Organ Failure Assessment (SOFA) and other illness severity scores are prognostic scores often adopted in ED for septic patients. However, validation of these scores as decision-making tool in ED for septic patients is lacking.

SOFA score discriminates septic patients at risk of disease progression and death better than previous “severe and inflammatory response syndrome” (SIRS) criteria in the Intensive Care Unit (ICU) [2]. This score has been developed in ICU setting and its usefulness outside ICU has not been well established. Also, the “quick” version of the SOFA score (qSOFA), a prognostic score used in ED [3], has shown low accuracy to predict mortality [4] and its performance can be lower than other prognostic scores commonly adopted in ED [5–7]. In particular, SOFA and qSOFA performance depend on age-related risk factors, such as comorbidities, which prevalence is high in patients seen in ED [8, 9]. Of note, the usefulness of qSOFA to diagnose sepsis in ED remained a matter of debate [10] and SIRS criteria are still adopted in some ED [4], despite of current indications [11].

Age-related risk factors are not included in the SOFA score. Therefore, high-comorbidity patients with a suspected infection at risk of progression to septic shock or death might not be early recognized [12]. Patients with advanced age are characterized by a high prevalence of chronic diseases and are prone to develop infections and sepsis [13]. These patients can represent over one third of the population seen in ED [14]. A potential solution to predict critical outcomes and early addressing low- and high-risk patients to the right intensity unit could be the integration of commonly used prognostic scores that include age-related risk factors.

The aim of this study was to compare the discriminant properties of several prognostic scores currently adopted in ICU and ED. In particular, we have focused on SOFA, Acute Physiology and Chronic Health Disease Classification System (APACHE) II [15], Simplified Acute Physiologic Score (SAPS) II [16], and National Early Warning Score (NEWS) 2 [17]. We hypothesized that combining discrimination properties of prognostic scores in the early triaging of septic patients might improve patients’ management in ED.

## Patients, material, and methods

### Study design

This study was designed as a single center retrospective study. We enrolled patients with SIRS criteria for sepsis between January 2017 and February 2020. Patients selected for this study were those consecutively admitted in ED of the “San Antonio Abate” secondary hospital of Tolmezzo, in the Northeast of Italy. The hospital serves a mountain area with a high prevalence of elderly. External patients with SIRS criteria were immediately treated or kept observed in ED. According to clinical conditions and the subjective judgment of the in-charge physician, patients were moved to another ward with a lower (internal medicine) or higher (ICU) intensity level of assistance or continued treatment in ED (sub-intensive unit) until clinical stabilization. All-cause mortality, independent of hospital staying, was assessed by checking electronic records until 30 days after ED admission.

We included patients of all sexes, with age ≥ 18 years, suspected infection in at least one body site, at least two of SIRS criteria verified in the first 24 h of observation. SIRS criteria consist in body temperature ≤ 36 °C or ≥ 38 °C, heart rate ≥ 90 beat per minute, respiratory rate ≥ 20 act per minute or carbon dioxide arterial partial pressure ≤ 32 mmHg or need of mechanical ventilation for respiratory failure, and white blood cells ≤4000 per ml or ≥ 12,000 per ml or over 10% of band neutrophils. We excluded patients without confirmed sepsis, pregnant women, patients with significant missing data, or deceased in less than 24 h of observation.

At admission, we collected information about general clinical characteristics, type and number of comorbidities, and the principal site of infection. We monitored general vital signs, performed general laboratory tests and arterial blood gas analysis, measured markers of inflammation and infection, and calculate the Glasgow Coma Scale (GCS). In patients with suspected infection, we performed blood, urine, and/or other site material collection for microbiological culture. Results of microbiological cultures became available at least 3-days after the initial collection. We measured urine output during the first 24–48 h. Legionella and pneumococcal urine antigens were tested in all patients. Prognostic scores were calculated with variables collected within the first 24 h after admission. Age-weighted Charlson Comorbidity Index (CCI) was calculated to account for comorbidities by adding 1 point to the original CCI score each decade above 40 years of age [18].

The primary outcome of the study was the occurrence of either in-hospital death or mechanical ventilation (which occurred first), within 7 days from ED admission. Since in our ED organization, patients were moved to ICU when mechanical ventilation is needed, we considered this primary outcome fundamental for deciding which patient to address in a low- or high-intensity care unit. We chose as secondary outcome all-cause 30-day mortality to confirm the safety of a decision-making based on scores cut-offs. A documented infection or a positive microbiological culture and at least two SIRS criteria verified within 24 h from admission confirmed sepsis. All suspected septic patients were managed according to guidelines [19].

The Institutional Review Board of the University of Udine approved the study protocol.

### Laboratory methods

Not-fasted venous blood samples were collected in patients with suspected sepsis at admission in ED. Blood gases, bicarbonate, hemoglobin, and electrolytes concentrations were available immediately by point-of-care analysis. Results of urgent venous blood exams were available usually within 1 h and those not-urgent in about 6 h after central laboratory submission. These results integrated inclusion criteria of patients with suspected sepsis and were used to calculate prognostic scores within the first 24 h. Red and white blood cells and platelets counts, hematocrit, hemoglobin, serum sodium, potassium, creatinine, blood urea nitrogen (BUN), and total bilirubin levels were measured in the centralized laboratory of the hospital by an automated analyzer. Microbiological cultures were processed and analyzed in the microbiological laboratory.

### Scores calculation

SOFA score was calculated by evaluating respiratory variables (ratio of oxygen partial pressure to the fraction of inspired oxygen P/F, arterial oxygen partial pressure, and need of respiratory support), coagulation (platelets count), liver function (total bilirubin levels), cardiovascular system (mean arterial blood pressure MAP and/or need of vasopressor support), central nervous system (GCS), and renal function (creatinine levels and urine output) [2]. NEWS2 was calculated by respiratory rate, arterial oxygen saturation, need of oxygen support, systolic blood pressure, heart rate, consciousness, and body temperature [17]. APACHE II score was calculated by acute physiological variables (body temperature, MAP, heart and respiratory rate, arterial oxygen partial pressure and pH, plasma bicarbonate, sodium, potassium, and creatinine, hematocrit, white blood cells, and GCS), age, and chronic health problems (hepatic, cardiovascular, respiratory, renal, or immunological diseases) [15]. SAPS II score was calculated by heart rate, systolic blood pressure, P/F, urine output, white blood cells, serum potassium, urea nitrogen, sodium, bicarbonate, bilirubin, GCS, age, chronic diseases (metastatic cancer, hematological malignancy, or acquired immunodeficiency syndrome AIDS), and type of admission (scheduled surgical, medical, or emergency surgical) [16].

### Statistics methods

Continuous normal variables were presented as mean ± standard deviation or, for skewed variables, as median and interquartile range [IQR]. Normal distribution of variables was assessed by Shapiro-Wilk test. Mean difference was analyzed with Student t-test or Mann-Whitney U test. The difference in proportions was analyzed with the Fisher exact test. Best cut-off value of each severity score for detecting primary outcome was determined by maximizing sensitivity and specificity values of the receiver operating characteristic (ROC) curve. For each cut-off value, we calculated relative metrics comprised sensitivity, specificity, and positive and negative predictive values with their respective 95% confidence interval (CI). The area under the ROC curve (AUROC) and its 95% CI were determined for each score to assess discrimination for the primary and secondary outcome. AUROC between prognostic scores was compared with De Long’s test, specificity and sensitivity with McNemar’s test, and positive and negative predictive values with a generalized score statistic. Survival probabilities have been presented with Kaplan-Meier curves and the log-rank test was used to compare 30-day survival probability above and below the cut-off points. Predictors of the primary outcome were determined with logistic regression analysis and results were reported as odds ratio (OR) and 95% CI. Predictors of secondary outcome were determined by Cox regression analysis and results reported as hazards ratio (HR) and 95% CI. The multivariate model for predicting the primary and secondary outcome was determined by variable selection according to a stepwise analysis based on the Akaike information criterion (AIC). The sample size of this study gives a power of 80% with a type I error of 0.050 to detect a difference as greater than 0.20 between paired AUROC. We considered significant a *p*-value < 0.050. Statistical analysis was performed with the free R software version 4.0.3 [20].

## Results

We identified 270 patients with inclusion criteria, 149 of which were excluded; thus, 121 patients were ultimately included in the analysis. Ninety-nine patients (82%) were elderly with age 65 years or older. Hypertension was the most prevalent comorbidity (65%) followed by diabetes (40%), chronic heart failure (33%), chronic kidney disease (30%), malignancy (29%), peripheral artery disease (29%), chronic liver disease (17%), coronary artery disease (17%), cerebrovascular disease (16%), obesity (13%), and chronic obstructive pulmonary disease (12%). Microbiological cultures of blood, urine, or other biological matrix were performed in 103 patients (85%), 64 of which (62%) was positive for germs growth. Prevalent germs isolated were *Escherichia coli (*44%), *Staphylococcus aureus* (16%), and *Enterococcus faecalis* (8%). The first four sites of infection detected were lung (42%), urinary (23%), gastrointestinal (17%), and soft tissues (10%). Prevalence of comorbidities, isolated germs, and sites of infection did not differ whether primary or secondary outcome occurred.

**Fig. 1:**
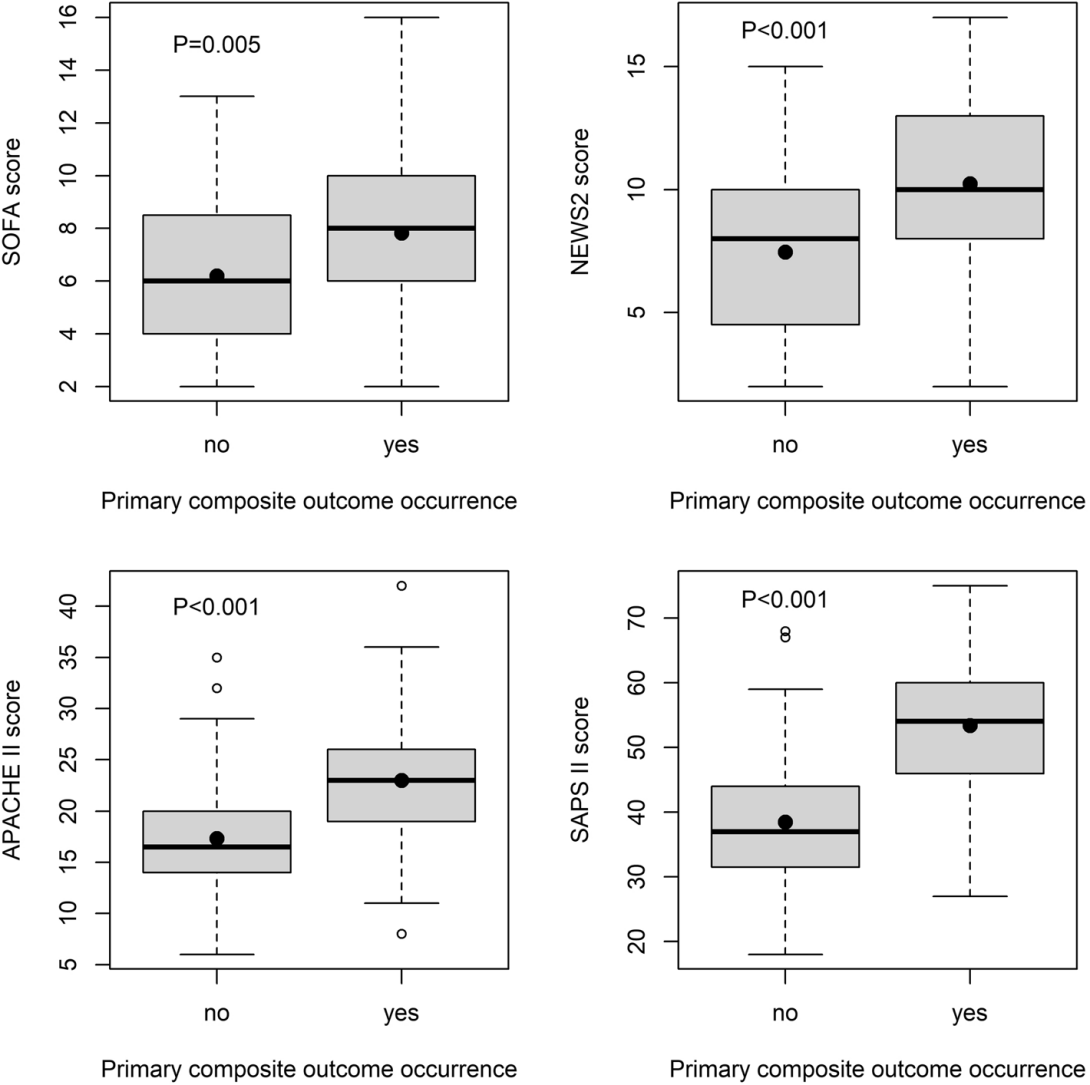
Box plots summarizing prognostic scores statistic according to the occurrence of the primary outcome. The plain black circle inside the box represents the mean value. P = probability of the Student’s t-test

The primary outcome occurred in 40% of patients. Specifically, in-hospital death within 7 days occurred in 28% and need of mechanical ventilation in 21% (Table 1). Median 30-day survival time of dead patients with primary outcome was 2 days (IQR 1–5), in those without outcome 12 days (IQR 9–17). Regarding patients without the primary outcome, those with the outcome had lower body temperature, GCS, MAP, arterial pH, and bicarbonate levels, and higher fraction of inspired oxygen, platelets count, sodium, and all prognostic scores (Table 1 and Fig. 1). Best predictive model for the primary outcome included the following variables: body temperature, GCS, MAP, heart rate, arterial oxygen saturation, white blood cells, platelets, serum sodium, and arterial pH. Independent negative predictors were MAP (each 1 mmHg, OR 1.964, 95% CI 0.936–0.990, *P* = 0.009) and arterial oxygen saturation (each 1%, OR 0.896, 95% CI 0.808–0.978, *P* = 0.023), whereas positive predictors were heart rate (each 1 bpm, OR 1.031, 95% CI 1.006–1.060, *P* = 0.021) and sodium levels (each 1 mmol/l, OR 1.126, 95% CI 1.043–1.229, *P* = 0.004). Forty-four percent of patients died within 30 days from admission with a 30-day median survival time of 4 days (IQR of 1 to 11).

**Table 1:** Comparison of variables collected at ED admission according to the primary outcome

	All patients	In-hospital death or need of mechanical ventilation within 7 days	Survived without need of mechanical ventilation	*P*-value
Generic variables				
Patients (n)	121	49	72	–
Age (years)	73 ± 10	75 ± 11	72 ± 10	0.056
Male sex [n(%)]	70 (58)	27 (60)	43(55)	0.708
Obesity [n(%)]	16 (13)	3 (6)	13 (18)	0.098
Charlson Comorbidity Index	5.7 ± 2.1	5.8 ± 1.9	5.6 ± 2.3	0.580
Body temperature (°C)	36.8 ± 1.3	36.2 ± 1.3	37.1 ± 1.3	< 0.001
Death within 7 days	34 (28)	34 (100)	0	–
Death within 30 days	53 (44)	39 (80)	14 (19)	< 0.001
Median survival time of dead patients (days)	4 [1–11]	2 [1–5]	12 [9–17]	< 0.001
Consciousness				
GCS	13.9 ± 2.4	13.0 ± 3.4	14.5 ± 1.0	0.004
Hemodynamic variables				
MAP (mm Hg)	71 ± 19	63 ± 15	77 ± 20	< 0.001
Heart rate (bpm)	101 ± 22	104 ± 22	99 ± 22	0.268
Respiratory variables				
Respiratory rate	24 ± 6	24 ± 6	23 ± 6	0.391
SpO_2_ (%)	95 ± 5	95 ± 6	96 ± 5	0.311
PaO_2_ (mm Hg)	89 ± 37	93 ± 47	86 ± 30	0.346
P/F ratio	272 ± 120	252 ± 126	285 ± 126	0.153
Need of mechanical ventilation [n(%)]	26 (21)	26 (100)	0	–
Other laboratory variables				
WBC (cells × 10^3^/mm^3^)	14.7 ± 8.0	14.6 ± 8.5	14.7 ± 7.6	0.933
Hematocrit (%)	35 ± 8	34 ± 8	35 ± 7	0.529
Platelets (cells ×10^3^/mm^3^)	210 ± 126	248 ± 140	185 ± 109	0.009
Serum sodium (mmol/l)	137 ± 7	139 ± 8	135 ± 5	0.002
Serum potassium (mmol/l)	4.31 ± 1.05	4.41 ± 1.18	4.24 ± 0.95	0.395
Arterial pH	7.35 ± 0.13	7.30 ± 0.15	7.38 ± 0.10	0.002
Plasma bicarbonate (mmol/l)	19 ± 7	17 ± 8	20 ± 6	0.028
Serum creatinine (mg/dl)	2.2 [1.2–4.2]	2.2 [1.4–4.3]	2.1 [1.2–4.1]	0.554
BUN (mg/dl)	42 [26–70]	48 [31–72]	37 [25–63]	0.183
Serum total bilirubin (mg/dl)	0.7 [0.5–1.6]	0.7 [0.6–1.6]	0.8 [0.5–1.6]	0.951

**Fig. 2:**
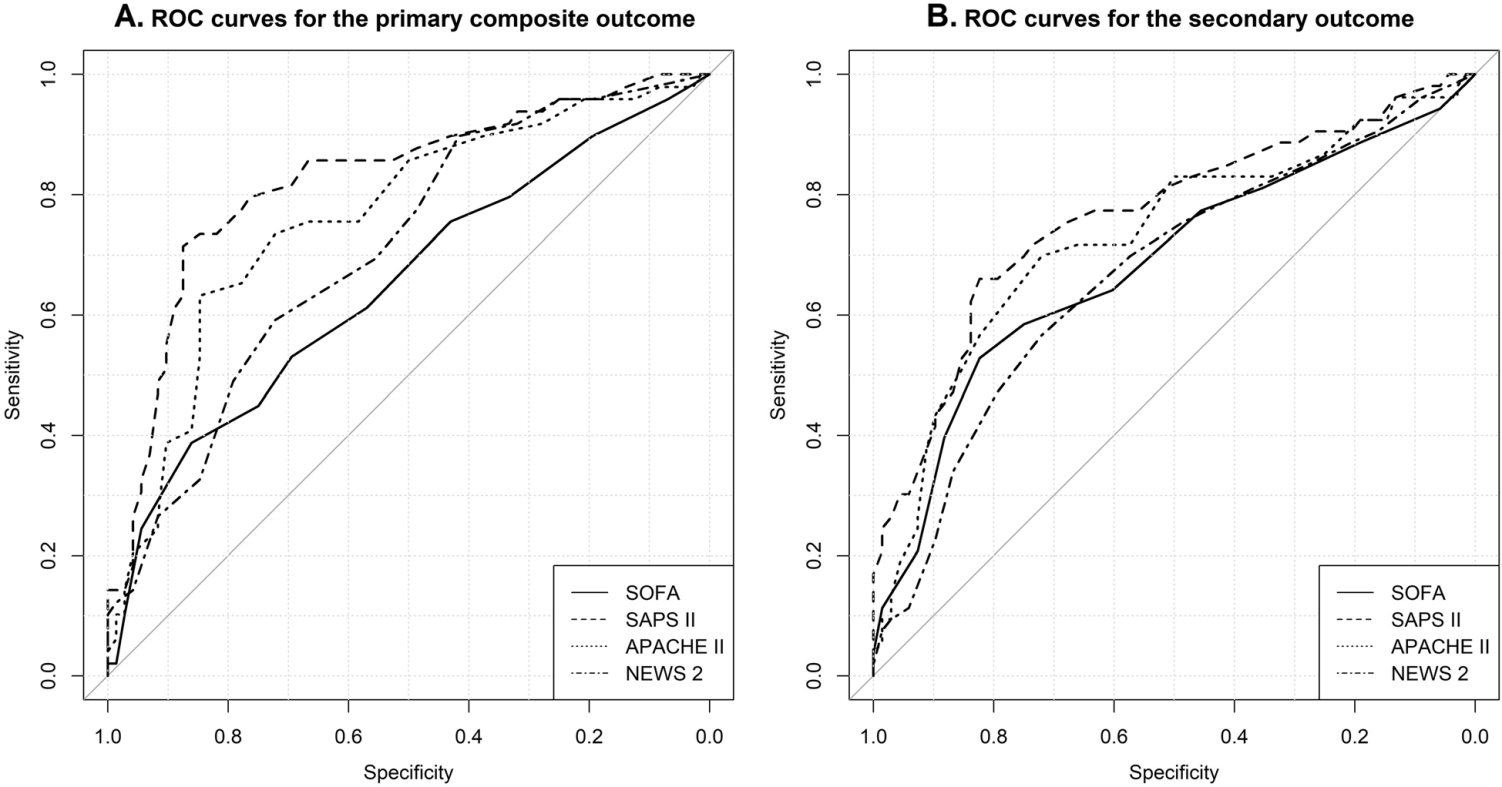
Receiver operative characteristic (ROC) curves of each severity score for predicting the primary composite (A) and secondary (B) outcome

The score with the best discrimination for the primary outcome based on AUROC was SAPS II, followed by APACHE II, NEWS2, and SOFA (Fig. 2A and Table 2). In Table 2, we report the best cut-off value that maximizes sensitivity and specificity of different scores and its relative metric for discriminating the primary outcome. SAPS II cut-off of 49, showed the lowest false positive rate (12, 95% CI 5–20) and the highest positive predictive value of 80% (95% CI 68–92). Median survival time of true-positive patients with SAPS II cut-off equal or above 49 was 2 days (IQR 1–5), need of mechanical ventilation in patients below 49 was 10%. NEWS2 cut-off of 7 showed the lowest false-negative rate (10, 95% CI 2–19) and the highest negative predictive value of 86% (95% CI 74–97). Median survival time of false-negative patients with NEWS2 cut-off below 7 was 9 days (IQR 4–11) and need of mechanical ventilation was 3%. Using SAPS II cut-off above 48 and NEWS2 below 7 for detecting high- and low-risk patients, respectively, classify 64% of patients within 7 days from admission, whereas 36% remained in a “gray zone”. In the “gray zone”, the primary outcome occurred in 26% of patients, median survival time of dead patients was 11 days (IQR 8–17), and need of mechanical ventilation 16%.

**Table 2:** Best cut-off values and relative metrics with 95% confidence interval based upon the receiver operating characteristic curve analysis for the primary outcome

Risk score	Best cutoff point	Sensitivity	Specificity	Positive predictive value	Negative predictive value	AUROC
SOFA	10	0.388 (0.251–0.524)	0.861 (0.781–0.941)	0.655 (0.482–0.828)	0.674 (0.578–0.770)	0.650 (0.548–0.751)
NEWS2	7	0.898 (0.813–0.983)	0.417 (0.303–0.531)	0.512 (0.406–0.617)	0.857 (0.741–0.973)	0.708 (0.616–0.800)
APACHE II	22	0.633 (0.498–0.768)	0.847 (0.764–0.930)	0.738 (0.605–0.871)	0.772 (0.680–0.865)	0.762 (0.673–0.850)
SAPS II	49	0.714 (0.588–0.841)	0.875 (0.799–0.951)	0.795 (0.676–0.915)	0.818 (0.732–0.904)	0.823 (0.744–0.902)
***P*-value for paired metric comparisons**						
SOFA-NEWS2	–	< 0.001	< 0.001	0.074	0.003	0.341
SOFA-APACHE II	–	0.003	0.781	0.315	0.005	0.002
SOFA-SAPS II	–	0.001	0.781	0.109	< 0.001	0.047
NEWS2-APACHE II	–	< 0.001	< 0.001	< 0.001	0.131	0.341
NEWS2-SAPS II	–	0.003	< 0.001	< 0.001	0.416	0.025
APACHE II-SAPS II	–	0.206	0.527	0.333	0.165	0.107

All prognostic scores predicted the secondary outcome. The best AUROC was that of SAPS II (0.757, 95% CI 0.659–0.855), followed by APACHE II (0.754, 95% CI 0.649–0.859), SOFA (0.691, 95% CI 0.591–0.792), and NEWS2 (0.681, 95% CI 0.573–0.788, Fig. 2B). Best independent negative predictors of 30-day mortality were body temperature, MAP, arterial oxygen saturation, and hematocrit level. Positive predictors were male sex, heart rate, and serum sodium level. In univariate analysis, GCS, platelets count, and need of mechanical ventilation were associated with 30-day mortality, whereas respiratory rate, oxygen arterial partial pressure, P/F ratio, arterial pH, plasma bicarbonate, white blood cells, creatinine, potassium, and total bilirubin levels were not (Table 3). When prognostic scores were included one by one in the best model, only SAPS II was an independent predictor of the secondary outcome (HR for each 1 point score 1.03, 95% CI 1.00–1.06, *P* = 0.047).

**Table 3:** Univariate analysis and multivariate best predictive model for 30-day mortality based on stepwise analysis according to the Akaike Information Criterion

	Univariate analysis		Multivariate best model	
	HR (95% CI)	P	HR (95% CI)	P
Age (each 1 year)	1.018 (0.990–1.048)	0.208	–	–
Male sex (yes/no)	1.164 (0.671–2.019)	0.589	1.809 (1.019–3.213)	0.043
Obesity (yes/no)	0.321 (0.100–1.031)	0.056	–	–
Charlson comorbidity index (each 1 point)	1.062 (0.942–1.197)	0.329	–	–
Body temperature (each 1 °C)	0.645 (0.513–0.812)	< 0.001	0.696 (0.551–0.879)	0.002
Glasgow coma scale (each 1 point)	0.875 (0.802–0.955)	0.003	–	–
MAP (each 1 mmHg)	0.967 (0.951–0.984)	< 0.001	0.971 (0.955–0.987)	< 0.001
Heart rate (each 1 bpm)	1.008 (0.995–1.020)	0.220	1.033 (1.018–1.048)	< 0.001
Respiratory rate (each 1 act/min)	1.016 (0.971–1.063)	0.497	–	–
SpO2 (each 1% point)	0.957 (0.915–1.000)	0.051	0.890 (0.845–0.938)	< 0.001
PaO2 (each 1 mmHg)	1.006 (0.999–1.013)	0.101	–	–
P/F ratio (each 1 point)	1.000 (0.997–1.002)	0.777	–	–
WBC (each 1000 cells/mm3)	0.993 (0.956–1.031)	0.712	–	–
Hematocrit (each 1% point)	0.977 (0.940–1.015)	0.230	0.954 (0.914–0.996)	0.032
Platelets (each 1000 cells/mm3)	1.002 (1.000–1.004)	0.018	–	–
Serum sodium (each 1 mmol/l)	1.057 (1.012–1.105)	0.013	1.051 (1.009–1.095)	0.017
Serum potassium (each 1 mmol/l)	1.171 (0.913–1.503)	0.215	–	–
Arterial pH (each 0.1 point)	0.888 (0.730–1.080)	0.234	–	–
Plasma bicarbonate (each 1 mmol/L)	0.969 (0.930–1.010)	0.129	–	–
Log [Serum creatinine (each 1 mg/dl)]	1.230 (0.876–1.726)	0.232	–	–
Log [BUN (each 1 mg/dl)]	1.001 (0.998–1.005)	0.442	1.483 (0.988–2.227)	0.057
Log [total bilirubin (each 1 mg/dl)]	1.264 (0.946–1.689)	0.113	–	–

**Fig. 3:**
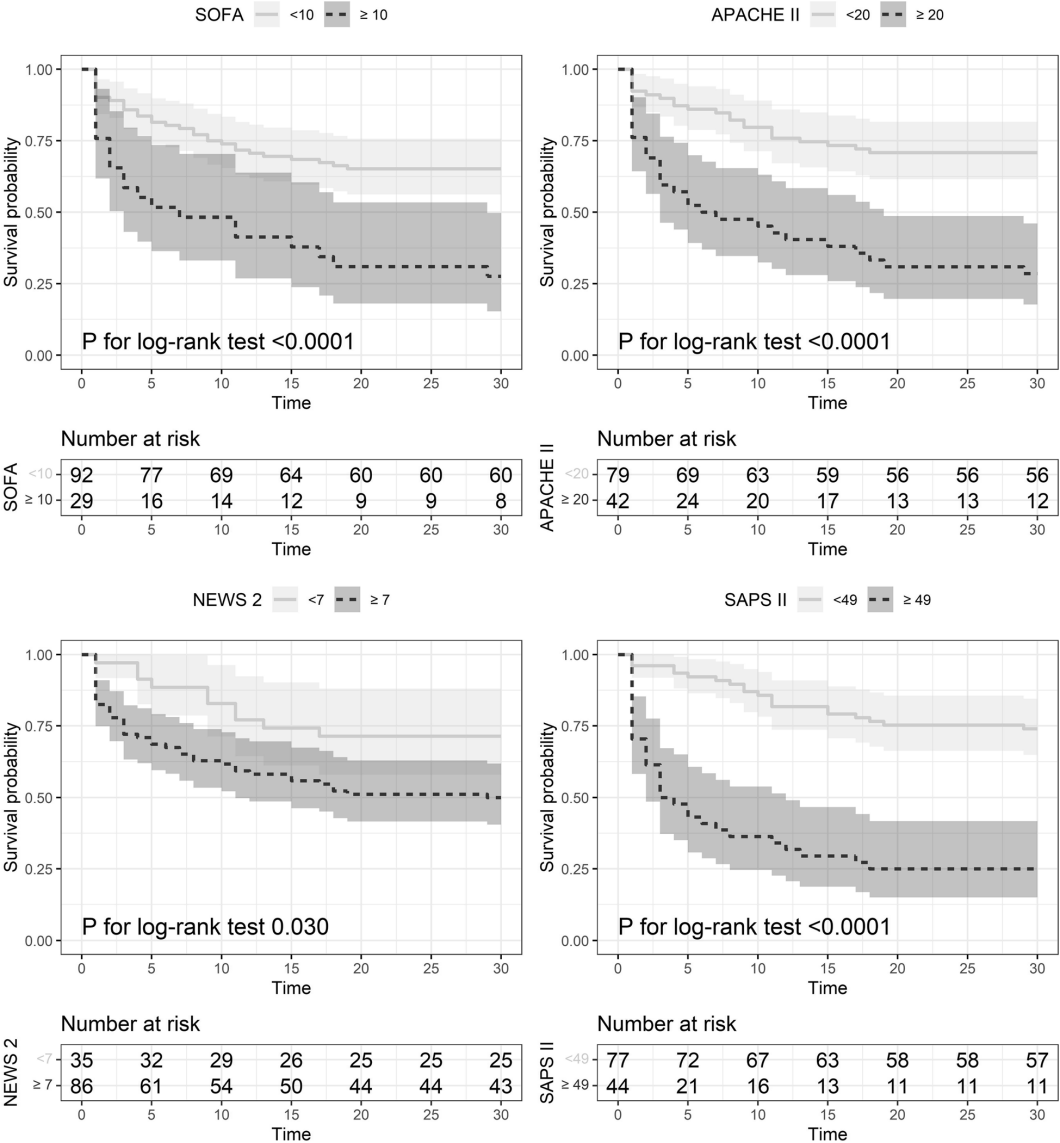
Kaplan-Meier curves representing survival probability of septic patients according to severity score above (continuous line) or below (dashed line) best cut-offs with respective 95% confidence interval (light and dark gray areas). Below plots, there are risk tables with number of survived patients at each time-point above or below the cut-off

In Fig. 3, we present the survival probability for 30-day mortality below and above the cut-off point for each score. All cut-off points well dichotomized patients in low- and high-risk of death within 30 days. SAPS II cut-off produced the highest difference in 30-day mortality (HR 5.24, 95% CI 2.99–9.21, *P* < 0.001).

## Discussion

In this study, SAPS II showed the best discrimination for both primary and secondary outcomes. Therefore, SAPS II score appeared the best candidate as a potential prognostic tool for early addressing septic patients to the more appropriate intensity level of assistance in ED. However, decision-making based only on this score is prone to misclassify negative patients that showed a high prevalence of mechanical ventilation. Using also NEWS2 score improved the selection of low-risk patients with a rate of false negatives more acceptable in low-intensive level units.

After the first 24 h of observation, addressing septic patients with SAPS II score above 48 to a sub- or high-intensive unit and those with NEWS2 score below 7 to a low-intensive unit appears safe and might reduce ED overcrowding. A workable solution for patients remaining in the “gray zone” may be implemented a sequential assessment of the prognostic scores across time, as suggested by Hwang et al. [21]. Since clinical conditions of septic patients can change rapidly [11], we suggest to updating prognostic scores every 24 h. According to our model, predictors of 30-day mortality to consider further for monitoring septic patients are body temperature, MAP, heart rate, arterial oxygen saturation, hematocrit, and serum sodium. These predictors could be implemented in existing scores for improving classification of “gray” patients, but this hypothesis should be verified in a large prospective study.

SAPS II score used in this study was validated in ICU and has shown only moderate predictive abilities when calculated with variables collected in ED [22]. A low performance outside the original development cohort is a common problem for the external applicability of prognostic scores. Therefore, scores models need to be recalibrated in different cohorts by redefining model parameters [23]. For risk classification based on a single cut-off, redefining the score model to improve discrimination performance means to determine new cut-off values or combining different predictors. In this study, we confirmed the moderate prognostic value of the single SAPS II score as observed in previous studies performed in ED [24, 25]. However, when we considered the performance of NEWS2 in low-risk patients combined with that of SAPS II in high-risk patients, we observed an increment in this prognostic value. In summary, instead of recalibrating scores models with new parameters, we propose to combine SAPS II and NEWS2 scores with new cut-off values. This maximizes true-positives and -negatives rates and permits to classifying correctly about two third of patients in the first 24 h after ED admission.

NEWS2 is widely used in ED as a prognostic tool [26, 27]. Engebretsen et al. showed that this score is a poor predictor of mortality, but a good predictor of ICU admission from ED [28]. In our ED organization, mechanical ventilation was a surrogate of ICU admission, since one implied the other. Therefore, having included mechanical ventilation in our primary outcome could be responsible for the moderate NEWS2 performance that we observed [28]. Accordingly, low-risk patients identified in our study by a NEWS2 score below 7 presented a low prevalence of primary outcome, mainly because of the low need of mechanical ventilation. This is an important point, since minimizing the risk of oral intubation is required for addressing patients safely to low-intensive units. Of note, despite of the lowest prevalence of mechanical ventilation in patients with NEWS2 below 7, the 30-day mortality of these patients was slightly higher than that of patients with SAPS II below 49. The relevance of this observation is not clear and should be assessed in further prospective studies.

SOFA has been developed as a prognostic score for septic patients in ICU [2], but its potential applicability for predicting mortality has been shown also in ED [24, 29]. In our study, SOFA score showed a poor discrimination ability and this discrepancy respect to previous studies should be explained. One reason could be associated with the age difference between studied cohorts. In particular, when SOFA was used to predict mortality in elderly patients with suspected infection, its discrimination ability worsen [30]. In addition, in old patients with sepsis, prediction of medium- and long-term mortality with SOFA was lower than scores that included age and other age-related risk factors [31, 32]. Also, SOFA performance improved when age and comorbidities were added to the prediction model for discriminating mortality outside ICU [12, 33]. In summary, discrimination performance of SOFA score seems dependent on age and age-related risk factors and it should be used cautiously in elderly patients.

This study has several limits to mention. First, it was a monocentric study and has been performed in a limited geographical area with a high prevalence of elderly. This location determined a selective cohort and a consequent relatively small sample size. Therefore, our results should be interpreted cautiously and should be limited to cohorts with similar characteristics. Second, this was a retrospective study and, by that, prone to selection bias. Also, uncontrolled confounders may have affected statistic associations by introducing potential mediator effects. To reduce these methodological problems, we adopted multivariate analysis with best model selection based on AIC and made our observations consistent by using confidence interval to report the effect size. Third, we are aware of the existence of other suitable prognostic scores that we could have tested in our ED cohort. However, prognostic scores adopted in this study were those currently available in our ICU and ED for managing septic and critical patients. Therefore, integrating these scores in our electronic ED system would be easier and practical for the everyday activity.

## Conclusions

SAPS II score discriminates against primary and secondary outcomes better than other prognostic scores. We suggest that patients with SIRS criteria for sepsis should be screened and treated within the first 24 h in ED and then addressed to low- or high-intensive care units according to SAPS II and NEWS2 scores cut-off values. Using SAPS II and NEWS2 scores to predict adverse outcomes and guiding decision-making in ED could be a novel approach for managing septic patients in secondary hospitals with a high prevalence of elderly or comorbidities.

## Data Availability

The datasets generated and analyzed during the current study are available from the corresponding author on reasonable request and after administrative authorization.

## Abbreviations

GCS: Glasgow coma scale
MAP: Mean arterial blood pressure
FiO_2_: Fraction of inspired oxygen
SpO_2_: Arterial oxygen saturation
PaO_2_: Arterial oxygen partial pressure
P/F: Ratio of oxygen partial pressure to the fraction of inspired oxygen
PaCO_2_: Arterial carbon dioxide partial pressure
BUN: Blood urea nitrogen
eGFR: Estimated glomerular filtration rate
WBC: White blood cells
SOFA: Sequential organ failure assessment
NEWS2: National early warning score 2
SAPS II: Simplified acute physiologic score II
ED: Emergency department
IRB: Internal review board
ICU: Intensive care unit
AUROC: Area under the receiver operating curve
AIC: Akaike information criterion
OR: Odds ratio
HR: Hazard ratio
CI: Confidence interval
